# Associations of cannabis and cigarette use with psychotic experiences at age 18: findings from the Avon Longitudinal Study of Parents and Children

**DOI:** 10.1017/S0033291714000531

**Published:** 2014-04-10

**Authors:** S. H. Gage, M. Hickman, J. Heron, M. R. Munafò, G. Lewis, J. Macleod, S. Zammit

**Affiliations:** 1 School of Social and Community Medicine, University of Bristol, UK; 2 MRC Integrative Epidemiology Unit, University of Bristol, UK; 3 UK Centre for Tobacco and Alcohol Studies and School of Experimental Psychology, University of Bristol, UK; 4 Institute of Psychological Medicine and Clinical Neurosciences, MRC Centre for Neuropsychiatric Genetics and Genomics, Cardiff University, UK

**Keywords:** ALSPAC, cannabis, psychosis

## Abstract

**Background.:**

A clearer understanding of the basis for the association between cannabis use and psychotic experiences (PEs) is required. Our aim was to examine the extent to which associations between cannabis and cigarette use and PEs are due to confounding.

**Method.:**

A cohort study of 1756 adolescents with data on cannabis use, cigarette use and PEs.

**Results.:**

Cannabis use and cigarette use at age 16 were both associated, to a similar degree, with PEs at age 18 [odds ratio (OR) 1.48, 95% confidence interval (CI) 1.18–1.86 for cannabis and OR 1.61, 95% CI 1.31–1.98 for cigarettes]. Adjustment for cigarette smoking frequency (OR 1.27, 95% CI 0.91–1.76) or other illicit drug use (OR 1.25, 95% CI 0.91–1.73) substantially attenuated the relationship between cannabis and PEs. The attenuation was to a lesser degree when cannabis use was adjusted for in the cigarette PE association (OR 1.42, 95% CI 1.05–1.92). However, almost all of the participants used cannabis with tobacco, including those who classed themselves as non-cigarette smokers.

**Conclusions.:**

Teasing out the effects of cannabis from tobacco is highly complex and may not have been dealt with adequately in studies to date, including this one. Complementary methods are required to robustly examine the independent effects of cannabis, tobacco and other illicit drugs on PEs.

## Background

Acute cannabis intoxication can cause transient psychotic experiences (PEs) (D'Souza *et al.* 2004) but it is less clear to what extent cannabis use leads to PEs not due to intoxication effects (Gage *et al.* 2013*b*). Longitudinal studies have attempted to assess this and a systematic review (Moore *et al.* 2007) reported consistent evidence of an association between cannabis and psychosis, but of modest size in epidemiological terms. Conclusions from other systematic reviews (Minozzi *et al.* 2010) and longitudinal studies (Callaghan *et al.* 2012; Rossler *et al.* 2012) are broadly consistent, although an earlier review reported inconsistent associations between cannabis use and psychological problems more broadly, and highlighted the need for stronger causal evidence, given the likelihood of residual confounding in most studies to date (Macleod *et al.* 2004).

In general, longitudinal studies controlling for more confounders (Zammit *et al.* 2002; Fergusson *et al.* 2005; Wiles *et al.* 2006) have reported point estimates that attenuate more than studies controlling for fewer confounders (Arseneault *et al.* 2002; Henquet *et al.* 2005). Important differences also exist in the extent to which studies adjust for tobacco and other illicit drugs. Tobacco use is strongly associated with psychosis (Morisano *et al.* 2009), and although evidence from animal models suggests possible mechanisms for smoking as a self-medication (Spielewoy & Markou, 2004; Weiss *et al.* 2007*a*, *b*) or for alleviating side-effects of antipsychotic medication, there is some evidence that people who smoke have an increased risk of developing later psychosis (Myles *et al.* 2012*b*). Tobacco use may be a marker for factors such as socio-economic status or family adversity that increase risk of psychosis (Hiscock *et al.* 2012), and that might confound the association between cannabis and psychotic outcomes. Of nine longitudinal studies examining this relationship to date, five attempted to adjust for tobacco use. However, the extent of adjustment varied, with three studies using binary measures of smoking and none using more detailed measures of smoking frequency. The relationship between cannabis, tobacco use and psychosis is complex and inadequately addressed to date (see, for example, van Gastel *et al.* 2013).

In this study we examined the relationships between cannabis, tobacco and PEs, attempting to take a more thorough approach to control for a wide range of potential confounders, including other substance use.

## Method

### Participants

The Avon Longitudinal Study of Parents and Children (ALSPAC) is a prospective, population-based birth cohort study that recruited 14 541 pregnant women resident in Avon, UK, with expected delivery dates from 1 April 1991 to 31 December 1992 (www.alspac.bris.ac.uk). There were 14 062 live births, and 13 988 infants survived to age 1 year. The current study is based on 4716 young people who completed the Psychosis-like Symptoms interview (PLIKSi) at age 18. The cohort has been described in detail previously (Boyd *et al.* 2013). Ethical approval was obtained from the ALSPAC Ethics and Law Committee and the Local Research Ethics Committee.

### Measures

#### Cannabis use

Data on cumulative cannabis use at age 16 were obtained (5068 participants responding) by a self-report questionnaire, and the responses were used to create a four-level category variable: ‘used 0 times’, ‘1–20 times’, ‘21–60 times’ and ‘more than 60 times’.

#### Cigarette use

The frequency of cigarette use at age 16 was also measured (5074 participants responding) by a self-report questionnaire, and the responses were used to create a four-level category variable: ‘non-smokers’, ‘experimenters’, ‘weekly smokers’ and ‘daily smokers’.

We also created a four-level composite measure of cigarette and cannabis use (low use of both, low-cannabis high-smoking, high-cannabis low-smoking, high use of both) using cut-offs of ‘more than 20 times’ and ‘weekly or more’ for high-cannabis and high-smoking respectively. The questions asked about cannabis and cigarette use are presented in the Appendix.

#### PEs

PEs were assessed at age 18 with a semi-structured interview (PLIKSi; Zammit *et al.* 2013) by trained psychologists. The PLIKSi has questions on 12 core experiences, including hallucinations (auditory and visual), delusions (for example being spied on or persecuted, having their thoughts read) and thought interference (broadcast, insertion or withdrawal), occurring in the past 6 months. Our primary outcome measure was a four-level category variable: ‘no PEs’, ‘suspected’, ‘definite’ and ‘psychotic disorder’. Participants were defined as having a psychotic disorder (Zammit *et al.* 2013) if they reported definite PEs, not attributed to sleep or fever, and occurring at least once a month for the previous 6 months. They also had to have caused severe distress, had a very negative impact on social or occupational functioning, or led to help-seeking from a professional. In the whole ALSPAC sample who completed the PLIKSi (*n* = 4716), more than 90% had no PEs, 4.3% had suspected PEs, 3.4% were rated as having definite PEs, and an additional 1.5% were rated as having psychotic disorder. In our complete case sample, the prevalence was lower, with 2.7% suspected, 1.9% definite and 0.9% psychotic disorder, as we excluded those rated as having definite PEs at age 12.

#### Confounders

Confounders considered, based on the literature for both cannabis and cigarette associations, were (*a*) pre-birth confounders (family history of schizophrenia or depression, maternal education, urban living, and gender), and (*b*) childhood confounders (IQ at age 8, borderline personality, bullying, peer problems, depression at age 12, and conduct disorder trajectory group ages 4–13). We also examined alcohol and other illicit drug use at age 16 as potential confounders. Cigarette and cannabis use were mutually adjusted for. Details are given in the Appendix.

### Statistical analysis

Analyses were conducted using Stata version 13 (StataCorp LP, USA). We assessed the relationship between cannabis/cigarette use and PEs before and after adjustment for confounders using ordinal logistic regression. We confirmed that the proportional odds/parallel regression assumption had not been violated using the Brant test. We examined the impact of the confounders on the association between cannabis use and PEs by comparing unadjusted estimates (model 1) with those adjusted for pre-birth confounders (model 2), and those further adjusted for childhood confounders (model 3). Further adjustment was made separately for cigarette or cannabis use as appropriate (model 4*a*), alcohol use (model 4*b*) and other illicit drug use (model 4*c*), all at age 16. Finally, we ran a fully adjusted analysis (model 5).

**Fig. 1.**
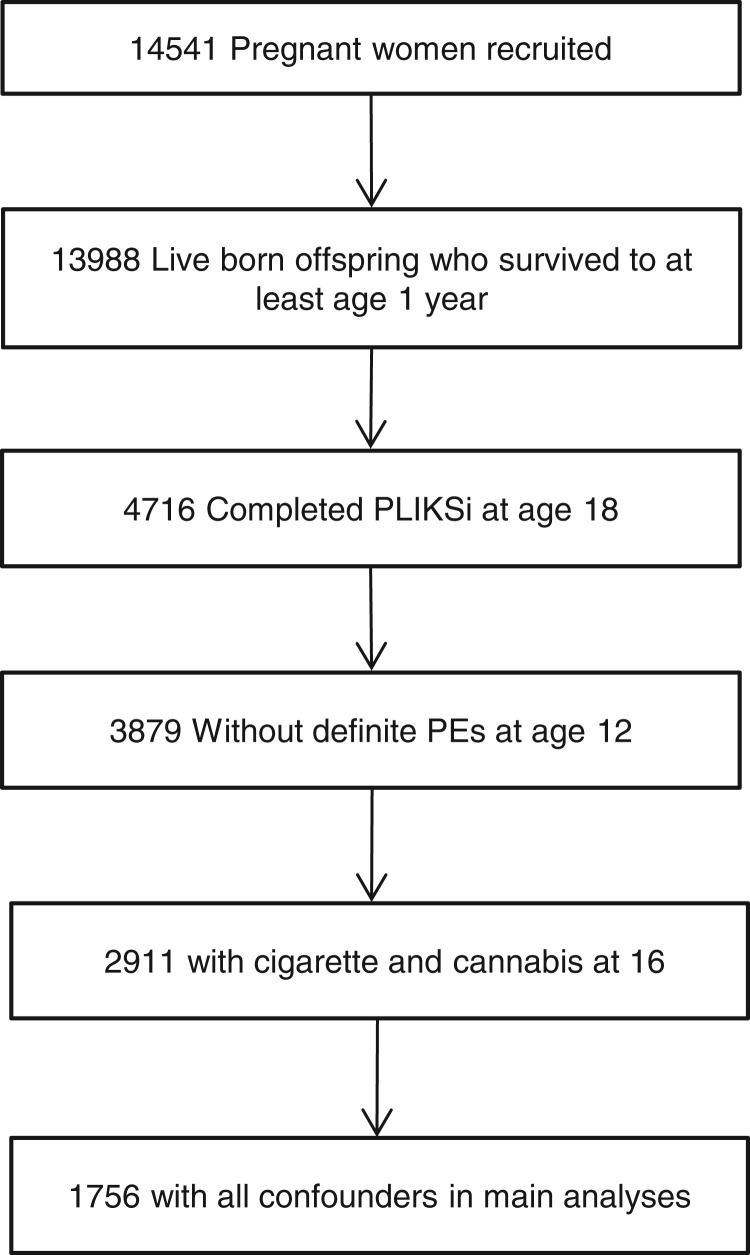
Study participant flow diagram.

As an attempt to minimize reverse causation effects, individuals who were judged to have definite PEs at interview at age 12 (*n* = 124; 77 definite PEs, 47 missing data) were omitted. The complete sample was 1756 (Fig. 1). A sensitivity analysis was also conducted, further excluding anyone who endorsed the definite presence of PEs on a self-report questionnaire at age 16. The complete sample for this analysis was 1573.

Participants were asked if their experiences only occurred within 2 h of using cannabis. As an additional step to exclude intoxication effects, analyses were repeated after excluding five participants who always attributed their PEs to cannabis intoxication.

To address potential bias from attrition, a multiple imputation (MI) analysis was undertaken, with 100 imputations. We used additional information from more than 50 variables associated with missingness and variables included in our analysis to make the missing-at-random assumption plausible, including measures related to pre-birth factors, and repeated measures of childhood and adolescent behaviours, self-reported psychotic-like experiences, and tobacco, cannabis and other substance use. MI is used as a sensitivity analysis, testing the robustness of findings in different samples. Analyses were repeated using imputed exposure and confounder data (imputed sample *n* =4484, after removal of those with definite PEs at age 12).

## Results

### Cannabis and PEs

Of the 1756 participants with complete data, 97 (5.5%) had incident suspected or definite PEs between ages 12 and 18. By age 16, 481 (27.4%) had ever used cannabis, with 57 (3.3%) having used cannabis more than 60 times. Cannabis use was more common in those who had: a family history of depression, mothers with higher education, higher IQ, childhood depression, early-onset persistent conduct disorder, and smoked cigarettes regularly (Table 1).

**Table 1.** Descriptives of confounders by cumulative cannabis use at age 16% (n)

Cumulative cannabis use age 16	0 times 72.1 (1275)	<20 times 20.4 (357)	21–60 times 4.1 (67)	>60 times 3.4 (57)	*p* value
Family history of depression	25.9 (330)	27.2 (97)	32.8 (22)	28.1 (16)	0.298
Family history of schizophrenia	0.8 (10)	1.1 (4)	0	1.8 (1)	0.625
No higher maternal education	76.8 (979)	69.2 (247)	62.7(42)	68.4 (39)	0.001
Urban dwelling	89.0 (1135)	86.8 (310)	89.6 (60)	86.0 (49)	0.353
Gender (female)	57.3 (731)	65.3 (233)	53.7 (36)	43.9 (25)	0.662
Borderline personality^a^	1.5 (19)	0.8 (3)	0	0	0.127
IQ at age 8 m(sd)	109.2 (15.4)	112.2 (15.2)	117.0 (14.2)	112.4 (13.8)	<0.001
Depression at age 12^b^	1.7 (22)	4.2 (15)	3.0 (2)	5.3 (3)	0.009
Early onset persistent conduct disorder group^c^	5.3 (68)	8.1 (29)	7.5 (5)	12.3 (7)	0.009
Peer problems (4 or more)	7.4 (94)	5.3 (19)	11.9 (8)	7.0 (4)	0.238
Bullied	30.3 (386)	29.7 (106)	29.9 (20)	28.1 (16)	0.712
Weekly or Daily cigarette smoking age 16	2.1 (27)	21.0 (75)	38.8 (26)	70.2 (40)	<0.001
Used other illicit drugs age 16	6.0 (76)	31.7 (113)	58.2 (39)	79.0 (45)	<0.001
Hazardous alcohol use age 16	20.9 (267)	63.0 (225)	61.2 (41)	86.0 (49)	<0.001

a Those with a score of 4 or over.

b Those with a score of 17 or over.

c As reported in Barker & Maughan (2009).

In the unadjusted analysis (Table 2), there was a 48% increase in odds across categories of PEs at age 18 per unit increase in cannabis use at age 16 [95% confidence interval (CI) 1.18–1.86]. This equates to a 3.2-fold increase in odds of PEs in adolescents who used cannabis more than 60 times compared to never users. This was essentially unchanged with adjustment for pre-birth or childhood confounders, or after omitting participants attributing their PEs to cannabis intoxication (data available on request).

**Table 2.** Ordinal logistic regression of cumulative cannabis use at age 16 and PEs at age 18

Model	*n* = 1756 (excluding PEs at 12)			*n* = 1573 (excluding PEs at 12 and 16)		
	OR	95% CI	*p*	OR	95% CI	*p*
1	1.48	1.18, 1.86	0.001	1.35	0.98, 1.86	0.070
2	1.53	1.21, 1.92	<0.001	1.40	1.01, 1.95	0.043
3	1.57	1.23, 2.00	<0.001	1.50	1.07, 2.10	0.019
4*a*	1.27	0.91, 1.76	0.160	1.08	0.69, 1.69	0.744
4*b*	1.57	1.19, 2.08	0.002	1.62	1.10, 2.39	0.015
4*c*	1.25	0.91, 1.73	0.165	1.30	0.84, 2.01	0.247
5	1.12	0.76, 1.65	0.553	1.09	0.65, 1.82	0.751

Model 1: PE at 18 by categorical cumulative cannabis use at 18.

Model 2: as model 1 with additional adjustment for pre birth confounders (family history of depression, family history of schizophrenia, gender, urban dwelling, maternal education).

Model 3: as model 2 with additional adjustment for childhood confounders (borderline personality, IQ at age 8, depression at age 12, conduct disorder trajectory group membership, peer problems, bullied).

Model 4*a*: as model 3 with additional adjustment for cigarette use.

Model 4*b*: as model 3 with additional adjustment for alcohol use.

Model 4*c*: as model 3 with additional adjustment for illicit drug use (other than cannabis).

Model 5: as model 3 with additional adjustment for cigarette, alcohol and other illicit drug use.

### Confounding by substance use

#### Cigarettes

Cigarette use and cannabis use at age 16 were highly correlated (polychoric *ρ* = 0.78, s.e. = 0.015). After adjustment for cigarette frequency, the relationship between cannabis use and PEs was attenuated by approximately 50% [adjusted odds ratio (OR) 1.27, 95% CI 0.91–1.76], equating to a 1.2-fold risk increase in those who used cannabis most heavily compared to never users. Adjusting for cigarettes using a less detailed measure reduced the impact of adjustment. Adjusting for binary measures of (i) dependence or (ii) ever *versus* never smoked (as used in many studies, for example: Fergusson *et al.* 2003; Henquet *et al.* 2005; Wiles *et al.* 2006; Rossler *et al.* 2012) resulted in adjusted ORs of 1.36 (95% CI 1.02–1.82) and 1.49 (95% CI 1.11–1.98) respectively.

Of 48 people who self-identified as using cannabis but not cigarettes, 46 responded to questions about how they smoked cannabis, and only 3 reported that they did not mix it with tobacco. As a result, although we were able to adjust for self-reported cigarette use (models 4*a* and 5), it was not possible to examine the relationship between cannabis and PEs independently of tobacco consumption.

#### Alcohol

In our sample, 65.5% of cannabis users reported excessive or hazardous drinking (*ρ* = 0.56, s.e. = 0.019). Adjustment for alcohol use did not attenuate the relationship between cannabis use and PEs.

#### Other drugs

Of those who had ever used cannabis, 41.0% used other drugs (*ρ* = 0.74, s.e. = 0.021) and 23.7% used stimulants. Adjustment for other drug use attenuated the relationship between cannabis use and PEs (adjusted OR 1.25, 95% CI 0.91–1.73). Because of the high correlation, we also investigated the association between cannabis and PEs in a restricted sample of 1483 participants who did not use other illicit drugs. There was no evidence of an association between cannabis and PEs in this restricted sample (Supplementary Table S1), and CIs were wider. However, the proportion of participants who used cannabis more than 60 times in this sample was considerably lower (0.8%) than in the full sample (3.3%).

### Cigarette use and PEs

Of the 1756 participants with complete data, 783 (44.6%) had ever used cigarettes and 91 (5.2%) smoked daily. Cigarette use at age 16 was more common in those with a family history of depression; who had mothers with no higher education; and who had a higher borderline score, depression, early-onset persistent conduct disorder, used cannabis, other illicit drugs or alcohol (Table 3).

**Table 3.** Descriptives of outcome and confounders by frequency of cigarette use at age 16% (n)

Freq of cigarette use age 16	Never 56.0 (973)	Experimenter 34.2 (615)	Weekly 4.1 (77)	Daily 5.7 (91)	*p* value
Family history of depression	25.2 (245)	28.1 (173)	23.4 (18)	31.9 (29)	0.175
Family history of schizophrenia	0.9 (9)	0.8 (5)	0	1.1 (1)	0.770
No higher maternal education	73.9 (719)	74.0 (455)	77.9 (60)	80.2 (73)	0.208
Urban dwelling	88.5 (861)	88.6 (545)	89.6 (69)	86.8 (79)	0.842
Gender (female)	51.1 (497)	67.5 (415)	71.4 (55)	63.7 (58)	<0.001
Borderline personality^a^	1.3 (13)	1.3 (8)	0	1.1 (1)	0.582
IQ at age 8 m(sd)	110.0 (15.4)	110.9 (15.3)	110.2 (13.7)	108.0 (15.9)	0.714
Depression at age 12^b^	2.0 (19)	2.8 (17)	3.9 (3)	3.3 (3)	0.169
Early onset persistent conduct disorder group^c^	5.3 (52)	6.7 (41)	7.8 (6)	11.0 (10)	0.024
Peer problems (4 or more)	7.4 (72)	6.7 (41)	1.3 (1)	12.1 (11)	0.859
Bullied	29.9 (291)	30.9 (190)	26.0 (20)	29.7 (27)	0.880
Ever used cannabis age 16	4.9 (48)	47.5 (292)	77.9 (60)	89.0 (81)	<0.001
Used other illicit drugs age 16	5.1 (50)	20.8 (128)	45.5 (35)	65.9 (60)	<0.001
Hazardous alcohol use age 16	15.1 (147)	51.2 (315)	70.1 (54)	72.5 (66)	<0.001

a Those with a score of 4 or over.

b Those with a score of 17 or over.

c As reported in Barker & Maughan (2009).

In the unadjusted analysis there was a 61% increase in odds of PEs per unit increase in cigarette use (95% CI 1.31–1.98), equating to a 4.2-fold increase in odds in those who smoked cigarettes daily compared to non-smokers. The association was attenuated slightly after adjustment for childhood confounders (Table 4).

**Table 4.** Ordinal logistic regression of cigarette use at age 16 and Psychotic Experiences at age 18

Model	*n* = 1756 (excluding PEs at 12)			*n* = 1573 (excluding PEs at 12 and 16)		
	Odds Ratio	95% CI	*p* value	Odds Ratio	95% CI	*p* value
1	1.61	1.31, 1.98	<0.001	1.62	1.23, 2.13	0.001
2	1.59	1.29, 1.96	<0.001	1.60	1.21, 2.13	0.001
3	1.53	1.23, 1.91	<0.001	1.63	1.22, 2.18	0.001
4*a*	1.42	1.05, 1.92	0.022	1.67	1.14, 2.45	0.008
4*b*	1.54	1.20, 1.98	0.001	1.85	1.32, 2.60	<0.001
4*c*	1.29	1.00, 1.68	0.051	1.52	1.09, 2.13	0.015
5	1.36	0.99, 1.86	0.055	1.77	1.18, 2.66	0.006

Model 1: PE at 18 by categorical frequency of cigarette use at 18.

Model 2: as model 1 with additional adjustment for pre birth confounders (family history of depression, maternal education).

Model 3: as model 2 with additional adjustment for childhood confounders (borderline personality, IQ at age 8, depression at age 12, conduct disorder trajectory group membership, peer problems, bullied).

Model 4*a*: as model 3 with additional adjustment for cannabis use.

Model 4*b*: as model 3 with additional adjustment for alcohol use.

Model 4*c*: as model 3 with additional adjustment for illicit drug use (other than cannabis).

Model 5: as model 3 with additional adjustment for cannabis, alcohol and other illicit drug use.

### Confounding by substance use

#### Cannabis

Adjustment for cannabis use further attenuated the relationship by approximately 30%, but the association persisted (adjusted OR 1.42, 95% CI 1.05–1.92), equating to a 2.9-fold increase in risk in daily smokers compared to non-smokers. A total of 359 participants used cigarettes but not cannabis.

#### Alcohol

Excessive or hazardous drinking was reported by 55.6% of cigarette users (*ρ* = 0.60, s.e. = 0.025). Adjustment for alcohol did not attenuate the relationship between cigarette use and PEs.

#### Other drugs

Of those who smoked cigarettes, 28.5% also used illicit drugs other than cannabis (*ρ* = 0.63, s.e. = 0.026) and 14.8% used stimulants. Adjustment for other drug use attenuated the relationship between cigarette smoking and PEs by approximately 40% (adjusted OR 1.29, 95% CI 1.00–1.68), with weaker evidence of an association between cigarette smoking and PEs, equating to a 2.5-fold increase in odds of PEs in daily cigarette smokers compared to non-smokers.

When using the composite measure of combined cannabis and cigarette use, there was strong evidence that the odds of PEs were increased in all categories compared to those with low use of both substances (Table 5) in models adjusting for pre-birth and childhood confounders, though evidence was weaker in models further adjusting for other illicit drug use.

Sensitivity analyses excluding individuals with PEs at 16 resulted in weaker evidence of associations between cannabis and PEs, but stronger evidence of associations between cigarette use and PEs.

**Table 5.** Ordinal logistic regression of combined cigarette and cannabis use at age 16 and Psychotic Experiences at age 18

	Low cigarette use, high cannabis use (*n* = 58)			High cigarette use, low cannabis use (*n* = 102)			High cigarette use, high cannabis use (*n* = 66)		
	OR	95% CI	*p*	OR	95% CI	*p*	OR	95% CI	*p*
1	2.50	1.04, 6.02	0.041	2.80	1.46, 5.34	0.002	3.73	1.83, 7.60	<0.001
2	2.76	1.13, 6.75	0.026	2.57	1.34, 4.95	0.005	3.87	1.89, 7.95	<0.001
3	3.41	1.38, 8.46	0.008	2.33	1.17, 4.63	0.016	3.77	1.78, 7.97	0.001
4*a*	n.a.	n.a.	n.a.	n.a.	n.a.	n.a.	n.a.	n.a.	n.a.
4*b*	3.47	1.36, 8.85	0.009	2.37	1.16, 4.85	0.018	3.86	1.72, 8.67	0.001
4*c*	2.55	0.96, 6.78	0.060	1.88	0.91, 3.87	0.088	2.42	0.97, 6.06	0.059
5	2.60	0.95, 7.11	0.062	2.58	0.99, 6.74	0.052	3.05	1.08, 8.61	0.035

Basline: low cigarette, low cannabis (*n* = 1530).

Model 1: PE at 18 by categorical cannabis and tobacco use at age 16 (excluding those with PEs at age 12).

Model 2: as model 1 with additional adjustment for pre birth confounders (family history of depression, family history of schizophrenia, gender, urban dwelling, maternal education).

Model 3: as model 2 with additional adjustment for childhood confounders (borderline personality, IQ at age 8, depression at age 12, conduct disorder trajectory group membership, peer problems, bullied).

Model 4*a*: n.a.

Model 4*b*: as model 3 with additional adjustment for alcohol use.

Model 4*c*: as model 3 with additional adjustment for illicit drug use (other than cannabis).

Model 5: as model 3 with additional adjustment for alcohol and other illicit drug use.

### MI

For cannabis and PEs, point estimates derived from the imputation were very similar to those from the complete-case analysis. In the cigarette use analyses, point estimates were closer to the null in the imputed models, and evidence of an association was weaker than in the CCA. However, the pattern of association was similar, with adjustment for cannabis use or other illicit drug use most strongly attenuating the association in the imputed dataset (Supplementary Tables S2 and S3).

## Discussion

Both cannabis and cigarette use were associated with later incident PEs. Adjustment for pre-birth and childhood confounders did not substantially change point estimates. Additional adjustment for other illicit drug use or cigarette use attenuated cannabis use associations to a slightly greater degree than adjustment for cannabis or other illicit drug use attenuated the relationship between cigarettes and PEs. After adjustment for cannabis, there was still a 2.9-fold increase in odds of PEs in daily smokers compared to non-smokers, whereas after adjustment for cigarettes the odds of PEs in the highest cannabis category was 1.2 times higher than in non-users, and CIs included the null.

Although a strength of this study is that we have taken a more robust approach to minimize confounding, using a broad range of confounders throughout child and adolescent development, we were limited in our ability to tease out the effects of cannabis use independently of tobacco use because of their co-occurrence. There are almost certainly shared genetic and environmental effects on tobacco and cannabis dependence (Agrawal *et al.* 2009, 2012), and this would contribute to difficulties in teasing out independent effects of these on PEs. Although standard errors suggested that collinearity was not undermining regression modelling, almost all cannabis users who reported not smoking cigarettes smoked cannabis with tobacco. If the biological effects of tobacco confound the relationship between cannabis and PEs, then our results are undermined by this misreporting of tobacco use (because people smoking tobacco with cannabis are classed as non-cigarette smokers). However, if tobacco use does not have a causal effect on PEs (and there is little evidence that it does), this issue is less important. Smoking cigarettes might be a marker for socio-economic and environmental factors associated with cannabis use and PEs, which might confound the relationship between them. We might expect that the effect for cannabis would be more greatly attenuated by smoking than the effect for smoking is attenuated by cannabis, given differences in social patterns of cannabis and cigarette use. The strength of the correlation between cannabis use and cigarette use, however, means there are difficulties in interpreting the effects of cannabis as being independent of cigarette use (Davey Smith & Phillips, 1992). Further attempts to address this issue using a composite measure of use are problematic because of the smaller numbers in subgroups and reduced frequency of heavy cannabis users in light smokers or non-smokers.

The recent meta-analysis by Myles *et al.* (2012*a*) found no association between tobacco use and age of onset of schizophrenia, in contrast to other reviews reporting associations between cannabis use and age of onset. However, smoking status was examined after onset, and therefore smoking onset post-illness onset (for example to negate the side-effects of medication) would attenuate the association with age of onset.

A limitation of our study is that we relied on self-reported cannabis and tobacco use, without being able to validate these responses. Differences in the degree of attenuation could also occur if cigarette smoking was more accurately reported than cannabis use; as a result, there could be greater regression dilution bias for effects of cannabis because there is more measurement error. Previous longitudinal studies that adjusted for tobacco use have not found such attenuation between cannabis and PEs (Henquet *et al.* 2005; Wiles *et al.* 2006; Rossler *et al.* 2012). However, those studies that report the measures used all adjusted for binary categorization of smoking. When we collapsed our four-level tobacco measure to binary measures (containing less information), the resulting adjustment led to reduced attenuation. Previous studies may therefore not have adequately adjusted for the confounding effects of tobacco use.

We observed reasonably consistent evidence of an association between cigarette use and PEs. However, unlike with cannabis, there is no evidence that tobacco causes PEs during intoxication, and indeed several studies indicate that a reverse causation (or self-medication) explanation for the association is more likely (for a review, see Kumari & Postma, 2005). There have been fewer longitudinal studies focusing on the relationship between tobacco use and psychosis compared to cannabis use, and these report an increased risk of psychosis in smokers (Weiser *et al.* 2004; Sorensen *et al.* 2011), although in one study this was reversed after adjustment (Zammit *et al.* 2003).

It is apparent that teasing out independent effects of tobacco and cannabis on incidence of PEs is a complex problem that is probably not adequately achieved in most studies to date. Most studies do not provide detailed information about the co-occurrence of these substances, and their findings need to be interpreted in the light that they may have suffered from similar limitations. Similar problems also exist when teasing out the effects of cannabis use on psychosis from that of other drugs. Although many cannabis users may not have used other illicit drugs, using restriction as a method of dealing with confounding leads to a loss of heavier cannabis users, as observed in our study.

### Implications

Although there is evidence that the relationship between cannabis and psychotic outcomes may not be solely due to intoxication effects (Kuepper *et al.* 2011), our findings highlight problems of disentangling confounding effects of other substances. In particular, associations of cannabis use *versus* cigarette use (or indeed nicotine) are rarely assessed in the same samples, and when they are, their effects are difficult to disentangle because of co-occurrence. Epidemiological evidence in support of a causal effect of cannabis on long-term risk of psychosis relies heavily on the clear evidence of increased risk of PEs occurring during intoxication. To improve our understanding of how cannabis and tobacco use impact on later PEs, a focus on areas of study where it is possible to examine long-term independent effects is needed (Hickman *et al.* 2009). This might include studies of long-term effects of tetrahydrocannabinol (THC) on mouse/rat brains (Malone *et al.* 2010). However, it might be possible to design more informative studies in humans. For example, studying populations where cannabis and tobacco are not usually used together might allow us to better investigate independent effects. Studying populations where confounding patterns are different in the different populations (Brion *et al.* 2011) would provide stronger evidence of a genuine association if it remains across both populations. Mendelian randomization studies, whereby genes or allelic risk scores are used as instrumental variables for cannabis use (e.g. Gage *et al.* 2013*a*) might be possible if genetic instruments or risk scores are developed. Long-term follow-up of randomized controlled trials that administer medical THC could also investigate PEs as a secondary outcome. It is through such approaches that a clearer understanding of the complex relationships between cannabis, tobacco, other drugs and psychosis may be achieved.

## Appendix

**Table:**

**Questions asked**
***Cannabis use***
How many times have you taken cannabis in total? Six options: ‘never’, ‘less than 5 times’, ‘5–20 times’, ‘21–60 times’, ‘61–100 times’ and ‘more than 100 times’
***Tobacco use***
Please mark the box that describes you best: Seven options: ‘I have never smoked a cigarette’, ‘I have only smoked a cigarette once or twice’, ‘I used to smoke sometimes but I never smoke cigarettes now’, ‘I sometimes smoke cigarettes but I smoke less than once a week’, ‘I usually smoke between 1 and 6 cigarettes a week’, ‘I usually smoke more than 6 cigarettes a week, but not every day’ or ‘I usually smoke one or more cigarettes every day’
***Confounders***
Family history of depression and schizophrenia: binary measures assessed by maternal questionnaire
Maternal education: a five-level categorical variable assessed by maternal questionnaire
Urban living: urban/town/village/hamlet, obtained from postcode of residence
IQ at age 8, assessed by the Wechsler Intelligence Scale for Children (WISC; Wechsler *et al.* 1992)
Borderline personality: an eight-level measure assessed by interview at age 11
Bullying: a two-level measure assessed by interview at age 8
Peer problems: a 10-point scale assessed by the Strengths and Difficulties Questionnaire (SDQ; Goodman, 1997) at age 8
Depression at age 12: a 26-point scale assessed by the Mood and Feelings Questionnaire (MFQ; Angold *et al.* 1995)
Conduct disorder trajectory group ages 4–13: membership of one of four trajectory paths (early onset persistent/childhood-limited/adolescent onset/low) as described by Barker & Maughan (2009)
Alcohol use: assessed using the Alcohol Use Disorders Identification Test (AUDIT; Saunders *et al.* 1993), score ranging from 0 to 40
Illicit drug use: a three-level measure (none/other drugs/stimulants) assessed by questionnaire
*Data sharing*: the full dataset and statistical code are available from the corresponding author. Participants gave informed consent for data sharing, full information on the ethical and governance stipulations around access to and sharing of ALSPAC data are available on the ALSPAC website (www.alspac.bris.ac.uk).

## Supplementary material

For supplementary material accompanying this paper visit http://dx.doi.org/10.1017/S0033291714000531.
